# Turkish version of the ‘Three-Factor Eating Questionnaire-51’ for obese individuals: a validity and reliability study

**DOI:** 10.1017/S1368980021000574

**Published:** 2021-08

**Authors:** Özge Küçükerdönmez, Rana Nagihan Akder, Selda Seçkiner, Esra Oksel, Şerife Akpınar, Eda Köksal

**Affiliations:** 1 Faculty of Health Sciences, Department of Nutrition and Dietetics, Ege University, İzmir 35100, Turkey; 2 Medical Faculty Hospital, Department of Endocrinology, Polyclinic, Ege University, İzmir, Turkey; 3 Faculty of Nursing, Department of Internal Medicine Nursing, Ege University, İzmir, Turkey; 4 Faculty of Health Sciences, Department of Nutrition and Dietetics, Gazi University, Ankara, Turkey

**Keywords:** Eating behaviour, Obesity, Reliability, Validity

## Abstract

**Objectives:**

Obesity is a serious public health issue. Investigating the eating behaviour of individuals plays an important role in preventing obesity. Therefore, the purpose of the current study is to adapt the long and first version of the ‘Three-Factor Eating Questionnaire’ (TFEQ), a scale that examines the eating behaviour of individuals, to Turkish culture and to carry out its validity and reliability study.

**Design:**

The data were collected using data collection forms, and anthropometric measurements of the individuals were made by the researchers. The data collection form included several parameters: socio-demographic characteristics, the TFEQ scale, whose validity and reliability analysis is conducted here, and the Dutch Eating Behaviour Questionnaire (DEBQ) which was used as a parallel form.

**Setting:**

The Obesity Clinic at Ege University in Izmir.

**Participants:**

The study group consisted of obese adult individuals (*n* 257).

**Results:**

It was seen that constructing the questionnaire with twenty-seven items and four sub-dimensions provides better information about Turkish obese individuals. Factor loadings ranged from 0·421 to 0·846, and item total score correlations ranged from 0·214 to 0·558. Cronbach’s *α* coefficient was found to be 0·639 for the whole scale. A positive, strong and statistically significant correlation was detected between TFEQ and DEBQ, which was used as a parallel form (*r* = 0·519, *P* < 0·001).

**Conclusion:**

In Turkey, the long version of the TFEQ scale was found valid and reliable for obese adult individuals. TFEQ can be used by clinicians or researchers to study the eating behaviour of obese individuals.

Obesity is simply defined as excess fat storage in the body. The prevalence of obesity has increased significantly in recent years and has become one of the serious global health problems. It is estimated that 1·5 billion of the global population will be overweight or obese by the year 2030^(1)^. In several studies, the prevalence of obesity in adult individuals in Turkey was found to be ranging between 22·3 and 30·3 %. In addition, based on the 2016 WHO data, Turkey was declared to possess the highest prevalence of obesity among European countries^(2,3)^. In epidemiological studies, obesity has been shown to be associated not only with type 2 diabetes, CHD, musculoskeletal diseases and some cancers but also with low self-esteem, depression and disability. In addition to the fact that it poses serious and life-threatening health problems for individuals, obesity also imposes a very high cost on society^(4,5)^. Non-communicable diseases rank first in terms of causes of death and disease burden in Turkey as well as in the rest of the world. The number of Disability Adjusted Life Years (DALY) that can be prevented by preventing obesity constitutes 7·3 % of the total DALY^(6)^. Therefore, investigation, treatment and prevention of obesity are of great importance.

The factors affecting obesity include age, gender, physical activity status, ethnicity, socio-economic status, genetic factors and eating behaviour^(4–7)^. The increasing prevalence of obesity in developed countries has developed the idea that high prevalence of obesity in certain populations is influenced by environmental factors as well as individual factors. As a matter of fact, the term ‘obesogenic environment’, which includes the common effect of environmental and behavioural factors, has started to be used frequently. It constitutes both the decrease in physical activity due to technology and the changes in eating behaviour (excessive consumption of simple sugars, fat, fast food and processed foods, reduction of fibre consumption, larger portions, differences in meal patterns)^(5,8,9)^.

Eating behaviour is a complex term that includes decisions made to questions such as ‘What to eat?’, ‘When to start eating?’, ‘When to stop eating?’ or ‘How much to eat?’. Internal factors (psychological status, genetic factors) and external factors (culture, socio-economic status, environmental factors) together play a role in making these decisions^(10–12)^. It is seen that the type of eating behaviour is determined based on the factors that cause overeating. For example, if excessive food consumption (overeating) occurs with loss of cognitive determination, it is called ‘restrained eating’, if it occurs when seeing or smelling delicious food, it is called ‘external eating’ and if it occurs due to a mood disorder, it is called ‘emotional eating’. In this context, each type of eating behaviour has a different aetiology, and while different eating behaviours can cause obesity, the opposite is true as well^(13,14)^.

The literature holds numerous scales that were developed to examine the eating behaviour of individuals. The Dutch Eating Behaviour Questionnaire (DEBQ), the Emotional Eating Scale, the Mindful Eating Questionnaire and the Three-Factor Eating Questionnaire (TFEQ) are some of these examples^(15)^. In subsequent studies, it is seen that the scale was created in three different forms: TFEQ-18, TFEQ-21 and TFEQ-51^(16–18)^. Studies from different countries have used these three different forms^(18–24)^. Although the validity and reliability studies for TFEQ-18 and TFEQ-21 were conducted in Turkey^(25,26)^, a validity and reliability study for the 51-item long and the first version of the scale in Turkey were not found. The aim of the current study is to adapt the TFEQ to Turkish language and culture, and to evaluate the validity and reliability of the scale based on the results of the study conducted on obese individuals.

## Method

### Study group

**Fig. 1:**
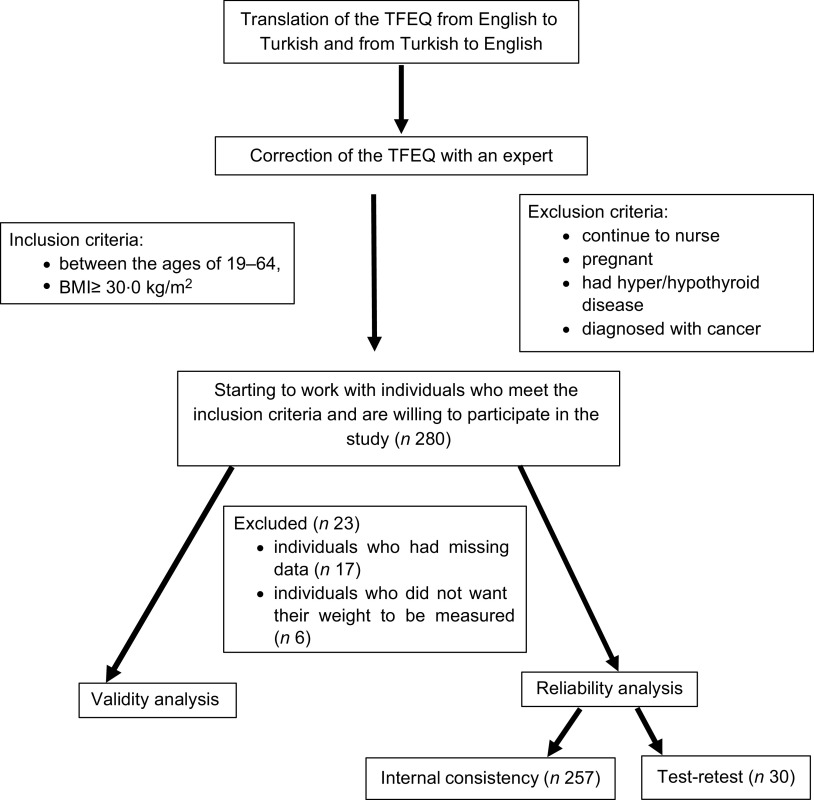
Study flow diagram

The study was carried out with individuals who applied to the Obesity Clinic at the Department of Endocrinology and Metabolic Diseases at Ege University. People between the ages of 19 and 64, with a BMI of 30·0 kg/m^2^ and above, were included in the study. The researchers performed the weight measurements using a Tanita MC780 device and height measurements using a stadiometer in accordance with their techniques^(27)^. Individuals who continue to nurse, are pregnant, had hyper/hypothyroid disease and were diagnosed with cancer or eating behaviour disorders were excluded. It was stated that the sample size should be 5–10 times the number of scale items in the validity and reliability studies^(28)^. Considering that the scale includes fifty-one items (51 × 5 = 255) and possible missing data (+10 %), 280 people were planned to participate in the study. The data were begun to be collected in January 2018 and when 280 people were reached (April 2018), the study was terminated. The study was completed with 257 individuals due to participants who had incomplete information in the data collection form (*n* 17) and did not want to get their weight measured (*n* 6) (Fig. 1). The Ethics Committee approval was obtained through applying to the Clinical Research Ethics Committee of Ege University (protocol number: 16-3.2/10).

### Data collection form

The data were collected in the Obesity Clinic at Ege University Hospital through face-to-face interviewing technique using data collection forms. These data were collected after the individuals were informed about the study. The data collection forms included socio-demographic characteristics (age, gender, marital status, educational status and occupation) (Table 1), anthropometric measurements (weight and height) and the TFEQ and the DEBQ scales. The administration of these interviews took 35–40 min.

**Table 1:** Socio-demographic characteristics of the individuals

Characteristics		*n*	%
Gender	Male	83	32·3
	Female	174	67·7
Level of education	Primary school and below	140	54·5
	Middle school	24	9·3
	High school	57	22·2
	Undergraduate and above	36	14·0
Marital status	Married	212	82·5
	Single	45	17·5
Occupation	Housewife	85	33·1
	Officer	14	5·4
	Private sector	40	15·6
	Self-employed	11	4·3
	Worker	67	26·1
	Retired	24	9·3
	Student	16	6·2
Total		257	100·0

The validity and reliability study of DEBQ in our country was conducted by Bozan *et al.* ^(29)^. The TFEQ was developed by Stunkard and Messic in 1985 and it included fifty-one items. The sub-dimensions of the scale, which also have three sub-dimensions, are as follows: (1) cognitive restriction of eating (conscious regulation of eating behaviours to maintain body weight within a healthy range), (2) not being able to restrict the eating behaviour – disinhibition (maintaining the eating behaviour even when not physiologically hungry) and (3) hunger (individual’s feeling of hunger and its effect on his/her eating behaviour). The scale does not have a total score and each sub-dimension is scored within itself^(17)^.

The TFEQ scale was translated from English to Turkish by two experts who spoke English well, and then from Turkish to English by another specialist. In line with the translations, a draft form was created taking the suggestions into account. The created draft form was presented for the opinions of ten faculty members working at the nutrition and dietetics departments of different universities. Following the expert opinions, necessary corrections were made again and the Turkish version of the scale to be tested was obtained. In order to determine the duration of implementation and to test the clarity of the data form, a pilot study was carried out with fifteen people who met the sampling criteria but whose data were not included in the current study. After encountering no problems in the pilot implementation, the actual study was commenced.

### Data analysis

The exploratory factor analysis was used to analyse the structural validity of the scale, and the confirmatory factor analysis was used to analyse the compatibility of sub-dimensions with the original scale. Before performing the exploratory factor analysis, the Kaiser–Meyer–Olkin test was applied to check whether the sample size was suitable for factor analysis. In order to reveal the factor pattern of the scale, principal component analysis was chosen as the factoring method, and varimax, a vertical rotation technique, was chosen as the rotation method. Items with factor loads below 0·30 or overlapping ones were removed from the scale. Item analysis for internal consistency was performed and the reliability coefficient (Cronbach’s *α*) was calculated.

The scale was repeated after an interval of 4 weeks for test–retest reliability. In addition, the DEBQ scale was used as a parallel form, and the relationship between the scale tested and the DEBQ scale was examined by Pearson correlation analysis. The SPSS v.25 software was used to evaluate the data, and the AMOS 21 programme was used for confirmatory factor analysis. The significance level was accepted as *P* < 0·05.

## Results

A total of 257 individuals, eighty-three males (32·3 %) and 174 females (67·7 %), with an average age of 43·11 ± 13·11 years, participated in the study. Approximately half of the individuals (54·5 %) were primary school graduates, and the vast majority (82·5 %) were married. When the distribution of the individuals participating in the research was examined according to their occupation, 33·1 % of the participants were housewives, 26·1 % were workers and 15·6 % were private sector employees. The vast majority of individuals (70·0 %) stated their income status as ‘income equal to expenditure’. The average BMI of the individuals participating in the study was 34·39 ± 4·66 kg/m^2^.

As a result of the analysis, the Kaiser–Meyer–Olkin value was found to be 0·821. In line with this result, it was concluded that the sample adequacy was ‘sufficient’ for factor analysis. In addition, when the results for the Bartlett’s test of sphericity were examined, it was seen that the *χ* ^2^ value obtained was acceptable (*χ* ^2^(351) = 1548·481; *P* < 0·05). According to the results of the exploratory factor analysis, it was found that there were twenty-seven items with a factor load above 0·30, while twenty-four items were removed from the scale. As a result of varimax rotation, the items were collected under a total of four factors. The names of the sub-dimensions and the minimum–maximum scores that can be obtained in each sub-dimension are as follows: uncontrolled eating (0–11), emotional eating (0–5), restrained eating (0–6) and conscious eating (0–5). These factors explain 41·336 % of the total variance. When the reliability of TFEQ and its sub-dimensions were evaluated separately, the reliability coefficients showed good reliability for uncontrolled eating (0·809), for emotional eating (0·619), for restrained eating (0·702), for conscious eating (0·606) and for the overall scale (0·639) (Table 2).

**Table 2:** Results of the exploratory factor analysis

	Factors				
Statements	F1: Uncontrolled eating	F2: Emotional eating	F3: Restrained eating	F4: Conscious eating	Total item correlation
S15	0·427				0·531
S24	0·438				0·480
S22	0·386				0·502
S26	0·437				0·484
S12	0·379				0·464
S34	0·477				0·487
S41	0·415				0·457
S1	0·357				0·448
S7	0·411				0·448
S19	0·389				0·418
S31	0·425				0·462
S20		0·649			0·558
S9		0·616			0·550
S27		0·532			0·444
S25		0·384			0·214
S11		0·341			0·251
S6			0·442		0·493
S32			0·435		0·368
S35			0·420		0·469
S28			0·417		0·463
S33			0·341		0·388
S18			0·427		0·419
S42				0·436	0·424
S44				0·415	0·372
S46				0·378	0·333
S48				0·359	0·378
S43				0·325	0·293
Reliability	0·809	0·619	0·702	0·606	0·639
Explained variance (%)	13·758	8·545	10·111	8·922	41·336

KMO = 0·821; *χ* ^2^(351) = 1548·481; Bartlett’s test of sphericity (*P*) < 0·001.

**Fig. 2:**
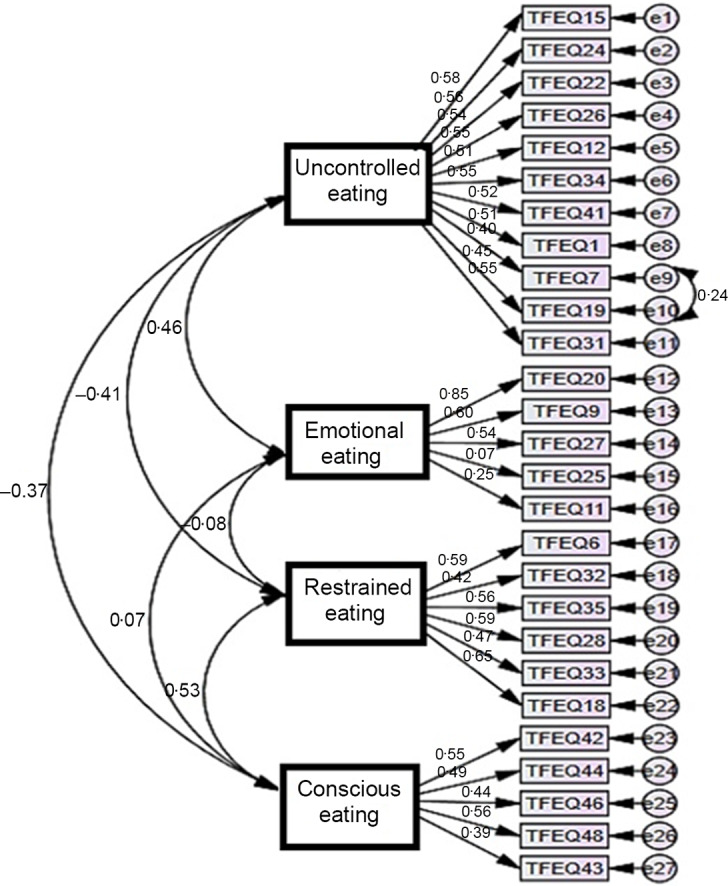
Modelling for the first-level multifactor confirmatory factor analysis

The independent samples *t* test results show the discriminative powers of all items. In order to determine the discriminatory features of the items in the scale, the raw scores obtained from each factor were ranked from low to high, and the average scores of the groups in the lower 27 % and the upper 27 % were compared using the independent samples *t* test. The results of the comparison showed that there was a significant difference between the averages of the sub and upper group item scores in terms of all items for each sub-dimension at *P* < 0·05 level. Modelling regarding the confirmatory factor analysis of the scale is shown in Fig. 2.

The reliability of the measurement model was tested by examining the average variance explained and compound reliability values of each factor separately. When the correlations between variables were examined, it was seen that the factor loads of the items were above 0·40 and all correlation relationships were significant (Table 3).

**Table 3:** Results of the measurement model

Factors	Statements	Parameter estimates (factor loadings)	*t* values	*P*	CR	AVE
F1: Uncontrolled eating	S15	0·585			0·81	0·71
	S24	0·549	6·898	*		
	S22	0·540	6·805	*		
	S26	0·555	6·947	*		
	S12	0·511	6·530	*		
	S34	0·552	6·923	*		
	S41	0·515	6·571	*		
	S1	0·508	6·504	*		
	S7	0·476	6·160	*		
	S19	0·447	5·854	*		
	S31	0·550	6·904	*		
F2: Emotional eating	S20	0·846			0·79	0·54
	S9	0·802	10·703	*		
	S27	0·544	8·104	*		
	S25	0·576	8·092	*		
	S11	0·449	3·653	*		
F3: Restrained eating	S6	0·594			0·70	0·56
	S32	0·421	5·183	*		
	S35	0·560	6·387	*		
	S28	0·587	6·581	*		
	S33	0·468	5·634	*		
	S18	0·551	6·321	*		
F4: Conscious eating	S42	0·549			0·73	0·46
	S44	0·487	5·071	*		
	S46	0·436	4·735	*		
	S48	0·558	5·442	*		
	S43	0·489	3·373	*		

* *P* < 0·001.

Some improvements have been made in the model. During this improvement, variables that reduce compliance were determined and a new covariance was created for the residuals with a high covariance. According to the confirmatory factor analysis, the scale was found to be significant at the level of *P* = 0·000 as a result of the structural equation model, and the twenty-seven items forming the scale were related to the four-dimensional scale structure (Table 4).

**Table 4:** Goodness of fit values of the structural model

	Structural model values	Acceptable values^(39)^
*χ* ^2^/df	1·346	< 5
RMSEA	0·037	< 0·08
GFI	0·890	≥ 0·80
AGFI	0·869	≥ 0·80
CFI	0·913	≥ 0·80

*χ* ^2^ = 426·810, df = 317, *P* < 0·001.

When the reliability of the DEBQ scale and its sub-dimensions were evaluated separately, the reliability coefficient was found as 0·852 for the ‘emotional eating’ sub-dimension, 0·852 for the ‘restrained eating’ sub-dimension, 0·857 for the ‘external eating’ sub-dimension and 0·896 for the overall scale. After the DEBQ scale was found to have a good reliability for the current study, its relationship with TFEQ was examined. There was a moderate positive and statistically significant relationship between the DEBQ scale and the TFEQ scale (*r* = 0·519, *P* < 0·05) (Table 5). A positive, statistically significant and strong correlation was observed between the test–retest total scores of the TFEQ scale (*r* = 0·966, *P* < 0·05) (Table 5).

**Table 5:** Correlations between TFEQ and DEBQ

	*r*	*P*
TFEQ and DEBQ	0·519	*
TFEQ and TFEQ a month later	0·966	*

* *P* < 0·001.

## Discussion

Assuming that eating behaviour consists of components such as emotional eating, uncontrolled eating, conscious eating, external eating and restrained eating, different scales have been developed to investigate these eating behaviours^(30,31)^. Among these scales, DEBQ and TFEQ are used extensively in studies on eating behaviour. Although the TFEQ scale was developed in 1985 with the purpose of to be used in obesity studies, its usability for obese individuals has not been tested for a long time^(16)^. From this perspective, we aimed to use the TFEQ scale in studies conducted with obese individuals in Turkey. Thanks to the validity and reliability studies conducted in different cultures and groups, we believe that these scales can measure different eating behaviour disorders or provide intercultural comparisons. As a result, it was found that the use of the TFEQ-51 scale for obese individuals in our country is valid and reliable. According to the exploratory factor analysis result, twenty-seven items with a factor load of 0·30 and above were included in the Turkish scale form. The Turkish form consisted of four sub-dimensions and the total variance ratio was 41·336 %. The Cronbach’s *α* coefficient calculated for the all dimensions (including sub-dimensions) of the scale was above the acceptable level (0·60). Moreover, there was a high correlation between the pre- and post-test results.

In multifactor patterns, an explained variance between 40 and 60 % is accepted as ‘sufficient’^(32)^. In our study, explained variance value of the four-factor scale was found to be 41·34 %, which corresponded with those from the literature. As a result of the analyses, it was seen that the scale with fifty-one items and three sub-dimensions in the original version would yield better results with twenty-seven items and four sub-dimensions for the Turkish culture. Similar to our study, in the study where the eighteen-item version of TFEQ was adapted to our country, four sub-dimensions were found to be more suitable^(26)^. While naming the sub-dimensions, they were presented for the opinions of experts (psychologist, psychiatrist and dietitian), the cases measured by the items were examined and it was decided that the sub-dimension names should be uncontrolled eating, emotional eating, restrained eating and conscious eating.

One of the methods in reliability analysis is the use of Cronbach’s *α* coefficient^(33)^. Cronbach’s *α* values > 0·60 indicate that the scales used are reliable^(34)^. The reliability coefficients of the sub-dimensions in the study ranged from 0·61 to 0·81. This shows that the scale used in the study has a good internal consistency. Another analysis in determining the internal consistency is item analysis^(35)^. In the current study, sub-dimension, total score correlation coefficients were calculated to be between 0·21 and 0·56. These items were not excluded from the scale since it was recommended to adhere to the original scale when Cronbach’s *α* value did not change with the removal of the item with a weak correlation. Another reliability criterion is the test–retest method^(36)^. In the current study, both pre- and post-correlation coefficients were 0·966. A high correlation between before and after the scale means that the scale is consistent over time.

As a result of the independent samples *t* test performed to determine the discriminative powers of the items, a significant difference was found between the upper 27 % and lower 27 % groups (*P* < 0·05)^(37,38)^. From this point of view, it can be said that the sub-dimensions of the scale are distinctive in the context of measuring the desired quality.

The composite reliability value of the implicit variables in the measurement model should be higher than 0·70, while the mean explained variance value should be higher than 0·50^(28)^. In our study, the compound reliability values were above the threshold value of 0·70, whereas only the average variance explained value of the ‘conscious eating’ factor (0·46) was below the threshold value of 0·50 in the measurement model. However, it has been reported that an average variance explained value of < 0·5 can be accepted when other reliability measurements are sufficient^(29)^.

The positive and strong correlation between the adapted scale (TFEQ) and the equivalent applied scale (DEBQ) means that the scale is validated. In the test–retest evaluation performed to measure the invariance of the scale over time, the correlation coefficient between the overall scale scores and the scale retest scores was 0·966 (*P* < 0·001). This result is of great importance in terms of showing the consistency of the scale over time.

## Conclusion

The results of our analyses evaluating the eating behaviours of obese individuals in Turkey showed that the TFEQ scale is a valid and reliable tool for individuals in this group. We believe that the underlying psychological causes of obesity can be better examined and problems can be resolved easier using this tool. In addition, it was seen that different versions of the scale have been used in different studies. In order to eliminate this confusion, we believe that researches in which different versions are compared or studies conducted with different sample groups are required.
